# Formation Dynamics of Honeycomb‐Like Capsules and Sponge‐Like Biofilms in *Micrococcus* Reveal a Novel Heavy Metal Avoidance Strategy

**DOI:** 10.1002/gch2.202500359

**Published:** 2025-11-17

**Authors:** Toshiyuki Kawamura, Yui Naito, Yosyun Onishi, Rajesree A/P Sivakumaran, Yuji Yanagihara, Eiki Matsui

**Affiliations:** ^1^ Department of Biology and Chemistry National Institute of Technology Fukui College Sabae City Fukui Japan; ^2^ Department of General Education National Institute of Technology Fukui College Sabae City Fukui Japan

**Keywords:** camphor tree, copper, honeycomb‐like Voronoi structure capsule, micrococcus, sponge‐like biofilm

## Abstract

It is unclear how *Micrococcus* adapts to leaf environments and why it exhibits exceptionally high heavy‐metal tolerance. Herein, we report that a *Micrococcus* strain isolated from camphor tree leaves forms previously undescribed sponge‐like biofilms and a capsule with a distinctive honeycomb‐like Voronoi structure. Capsule formation begins during cell division, wherein thin fibers appear between two dividing cells and gradually thicken to form a dense capsule. The capsule surface is densely perforated with cavities (95.0 ± 4.41 nm in diameter and 166.3 ± 5.91 nm in depth), resembling a Voronoi diagram. As the structure matures, filamentous connections between cells form sponge‐like biofilms. Energy‐dispersive X‐ray spectroscopy analysis revealed that these architectures retain essential metal ions, while limiting the uptake of toxic copper ions. These structures represent a novel defense strategy, distinct from conventional mechanisms in which heavy metals are directly adsorbed into cells or capsules. This structural strategy supports copper resistance and ecological adaptation on camphor tree leaves, where microorganisms encounter nutrient limitations and fluctuating moisture. Building upon these insights, our findings expand current understanding of microbial survival strategies, shows the importance of structural biology in the phyllosphere, and indicates potential applications in bioremediation.

## Graphical abstract

Capsular and biofilm structures in *Micrococcus* isolated from camphor trees exhibit sponge‐like and Voronoi‐patterned morphologies. These unique architectures are formed during cell division and may function in selective transport and defense, revealing a novel adaptation mechanism distinct from conventional metal adsorption strategies.

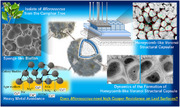

## 1 Introduction

*Micrococcus* spp. are widely distributed bacteria with notable ecological and environmental significance. In addition to their ecological roles in metal resistance [1, 2, 3, 4], *Micrococcus* spp. have been associated with plant‐related environments, where they may promote plant growth and stress tolerance [5, 6, 7]. Revealing their potential ecological relevance in such plant‐associated settings provides important context for understanding the study objectives. *Micrococcus* spp. were first isolated from human skin in the 1970s [8, 9], and are an essential component of the natural human microflora. In addition to their role as commensals, these bacteria can persist and function across diverse environments, ranging from natural ecosystems to heavily contaminated sites. As heavy metal‐resistant bacteria, *Micrococcus spp*. have also been isolated from aquatic environments [1, 2], forests, peri‐vegetative soils [5, 10], copper mines (*Micrococcus yunnanensis* GKSM13) [3], industrial effluents (*Micrococcus luteus* AS2 strain) [11], and mining sites (*Micrococcus* sp. BirBP01) [12]. For instance, *Micrococcus luteus* DE2008, isolated from the Ebro River delta, can grow under conditions of high lead and copper concentrations [1].

In addition, *Micrococcus* spp. play an active ecological role by removing heavy metal contamination through biosorption, as shown in several studies. Letnik et al. used scanning electron microscopy with energy‐dispersive X‐ray spectroscopy (SEM/EDS) and transmission electron microscopy (TEM) to assess the adsorption of copper by *M. luteus* in aqueous environments, revealing copper enrichment on the bacterial surface and intracellularly [4]. Moreover, Maldonado et al. reported that in contaminated cultures, *Micrococcus luteus* DE2008 effectively biosorbs extracellular lead and copper without intracellular accumulation [2]. Majhi et al. achieved a maximum copper ion (Cu^2+^) removal rate of 89.2% in *Micrococcus yunnanensis* GKSM13 cultures. Specifically, the authors reported the involvement of hydroxyl, carboxylamide, and amine groups in cell surface modification, Cu^2+^ deposition into bacterial cells, and Cu^2+^ removal using microscopy and spectral analysis [3]. This high resistance to copper and other heavy metals is attributed to the ability of *Micrococcus* spp. to bind metals with extracellular polysaccharides and internalize them. This resistance is also supported by active intracellular transport systems.

Mechanistically, adenosine triphosphate (ATP)‐binding cassette (ABC) transporters facilitate the in‐and‐out transport of copper in cells using ATP hydrolysis as an energy source [13]. The *CopA* and *CopB* efflux systems are examples of these ATPase transporters [14]. The *CopZ* family of copper‐binding proteins sequesters copper ions by transporting them to the appropriate intracellular locations for uptake and efflux by other copper‐binding proteins [15]. These molecular mechanisms are increasingly well understood; however, the higher‐order structural strategies, such as capsules and biofilms, which may also contribute to heavy metal resistance, remain unclear.

Because of the growing concerns over environmental pollution and the urgent need for sustainable remediation strategies, understanding the mechanisms through which *Micrococcus* spp. resist and remove heavy metals is highly relevant. These insights advance our knowledge of microbial adaptation and highlight the potential of *Micrococcus* spp. as contributors to sustainable bioremediation.

In plant‐associated environments, the ecological roles of *Micrococcus* extend beyond metal resistance. For instance, *Micrococcus* sp. NII‐0909 in forest soils is an effective biofertilizer that promotes plant growth through phosphate solubilization and indoleacetic acid (IAA) production [5]. The ability of *Micrococcus aloeverae* DCB‐20 to produce IAA without requiring tryptophan [6], indicates a close link between *Micrococcus* and plants.

*Micrococcus luteus* MlS14 promotes growth and stress tolerance in *Arabidopsis thaliana* by producing IAA and carotenoids [7]. Therefore, *Micrococcus* play important roles in environmental remediation and agriculture. The strong heavy metal resistance of *Micrococcus* spp. has been extensively investigated; however, most previous studies relied on liquid culture media or contaminated water [1, 3, 4]. To address this limitation in the present study, we aimed to elucidate the detailed morphology and copper resistance mechanisms of *Micrococcus* spp. isolated from camphor tree leaves and discuss their potential ecological relevance in plant‐associated environments. We focused on the higher‐order structures of biofilms formed on solid media, which closely mimic plant surfaces, as these structures provide insights into bacterial defense mechanisms that cannot be elucidated by investigating single cells.

Previous studies have revealed the functional role of biofilm structures in heavy metal resistance. For example, Teitzel and Parsek investigated the effects of heavy metals (copper, lead, and zinc) on *Pseudomonas aeruginosa* in biofilm and planktonic states. Biofilms exhibited resistance levels ranging 2–600‐fold higher than those of planktonic cells [16]. Furthermore, Stewart reported that metal ions move within biofilms by diffusion, based on Fick's law of diffusion, and showed that the time required for 90% (*t*_90_) of the metal ion concentration to reach the bottom or center of a flat biofilm can be calculated using this equation *t*_90_ = 1.03 × L^2^/De, where De is the effective diffusion coefficient within the biofilm and L is the distance. This indicates that the diffusion time significantly increases with distance, being proportional to the square of the distance [17]. Based on these reports, we hypothesized that the physical structure of biofilms plays an important role in bacterial heavy metal resistance, and that elucidating its structural details would contribute to understanding the exceptional copper resistance observed in *Micrococcus*.

Biofilm and capsule structures are largely dependent on extracellular polysaccharides. In Gram‐positive bacteria, capsular and extracellular polysaccharides are synthesized intracellularly and transported to the periplasm and extracellular space by flippase, subsequently forming the capsule layer [18, 19, 20]. This occurs through two primary pathways: 1) the Wzx/Wzy‐dependent pathway [21], in which short glycans released to the cell surface by the Wzx flipper are extended by Wzy polymerase, and 2) the ABC transporter‐dependent pathway [22], wherein glycans are assembled in the cytoplasm and transported by ABC transporters. Furthermore, capsular polysaccharide synthesis is closely linked to bacterial metabolism and pathogenicity [23, 24]. However, the relationship of these general insights with the persistence of phyllosphere‐dwelling bacteria, including *Micrococcus*, remains unclear.

*Micrococcus* are frequently found on plant surfaces; however, the structural systems that enable their persistence in such environments remain poorly understood. Structural information on their capsule and biofilm has also been limited. In this study, we investigated the detailed structures of the capsule and biofilm using SEM and TEM and discovered that during cell division, fibrous materials appear at the interface between dividing cells and form the capsule structure. Our findings also reveal novel insights into the structure of the capsule and biofilm. In addition, we observed an array of polygon‐shaped depressions on the capsule surface, which we considered to resemble a Voronoi‐like structure.

A Voronoi diagram is a spatial data interpolation method that divides the region nearest to each point into several regions on a plane. The boundary of each region is shaped by the midline to the neighboring point, forming a polygon [25]. The Voronoi structure can be applied to cell distribution, as previously observed in the microstructure of skeletal muscle [26], and plant stem tissue cross‐sections [27]. In addition, Voronoi's law may represent a form of evolutionary convergence that enables organisms to achieve strength and flexibility, as well as complex functions, such as mass transport.

In this study, we observed microscopic honeycomb‐like depressions on the surface of the capsular layer of *Micrococcus* sp. clone 20, isolated from the camphor tree, and determined that these structures had a Voronoi‐like pattern. The discovery of honeycomb‐like Voronoi structures on the capsule surface reveals a structural strategy that contributes to mechanical stability and controlled diffusion. This discovery indicates a novel structural strategy that supports the persistence of phyllosphere‐dwelling *Micrococcus* under environmental stress, such as heavy metal exposure, providing new insights into bacterial defense mechanisms and their ecological significance.

Therefore, this study provides novel structural and ecological insights into the means through which *Micrococcus* spp. persist under environmental stress, highlighting their potential importance in sustainable bioremediation and plant‐associated ecosystems.

## 2 Results

### 2.1 Identification of Bacteria Isolated from the Camphor Tree

**FIGURE 1:**
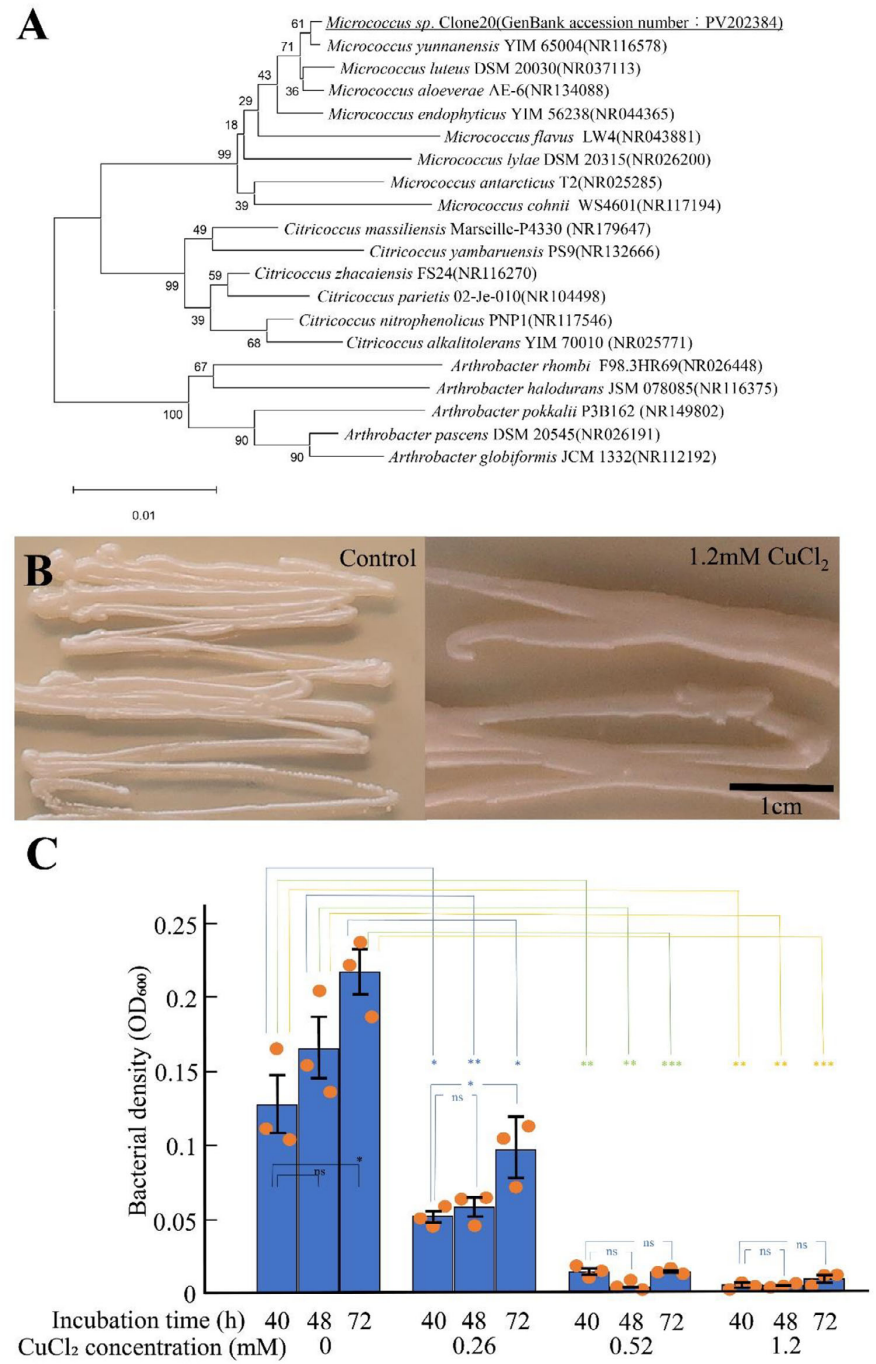
Phylogenetic analysis and copper tolerance assays of *Micrococcus* sp. strain clone 20 (Accession Number: PV202384) isolated from *Cinnamomum camphora*. (A) Phylogenetic tree constructed using the minimum evolution method (neighbor‐joining) based on 16S rDNA sequences with the Kimura 2‐parameter model. Bootstrap values were obtained from 1000 replicates. The scale bar represents 0.01 substitutions per site. (B) Copper tolerance assay on LB agar plates with cells retaining a biofilm‐morphology model. (C) Copper tolerance assay under shaken liquid culture conditions following vortex treatment, thus representing a nonbiofilm, planktonic‐cell model. Cell density was estimated by measuring optical density at 600 nm (OD_600_). Data are presented as mean ± SE (*n* = 3 independent experiments). Statistical significance was evaluated using a two‐tailed Student's *t*‐test assuming equal variance. ns, *p* ≥ 0.05; ^*^ *p* < 0.05; ^**^ *p* < 0.01; ^***^ *p* < 0.001.

Molecular phylogenetic analysis using 16S rDNA revealed that the bacteria isolated from camphor leaves belonged to the genus *Micrococcus*, which is one of three clusters: *Micrococcus*, *Citricoccu*s, and *Arthrobacter*. The isolate was designated *Micrococcus* sp. Clone20 (GenBank accession number: PV202384) (Figure 1A). The phylogenetic tree was constructed using the minimum evolution method (neighbor‐joining) and the Kimura 2‐parameter model, and bootstrap values were obtained from 1000 replicates (Figure 1A). However, the low bootstrap values hindered species identification in this phylogenetic analysis.

### 2.2 Comparison of Copper Tolerance Between Biofilm‐Forming Colonies on Agar and Planktonic Cells Released into Liquid Culture

On agar plates, colonies were selected using a sterile stick and gently spread across the surface of the medium to preserve the biofilm structure to some extent. Under these conditions, the addition of 1.2 mm copper chloride did not inhibit cell growth (Figure 1B). In contrast, when colonies were suspended in a liquid medium through vortexing and subsequently shaken to disrupt the biofilm structure and generate planktonic cells, cell density was monitored by measuring OD_600_. Data from three independent experiments are presented as mean ± SE. Statistical significance was assessed using Student's *t*‐test (two‐tailed), and the significance levels are shown in Figure 1B,C. Consequently, growth was completely inhibited following treatment with 0.52 and 1.2 mm copper chloride, whereas treatment with 0.26 mm copper chloride noticeably suppressed growth (Figure 1C). These results indicate that cells in a biofilm‐associated state exhibit higher copper tolerance than their planktonic counterparts.

### 2.3 Structure of Cells and Biofilm (Envelope) Secretions

**FIGURE 2:**
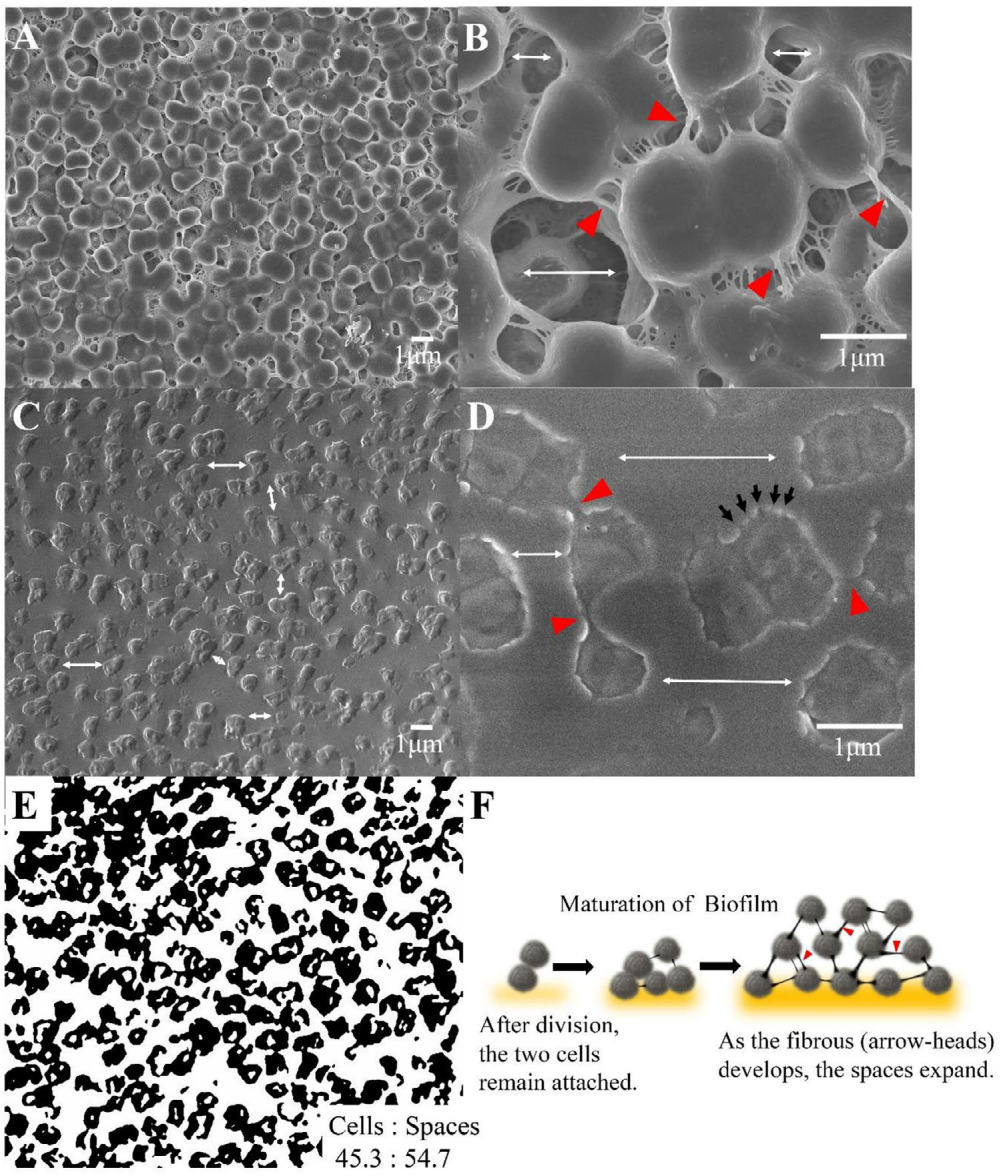
Sponge‐like structure of the biofilm surface and internal cross‐section observed using SEM. A) and (B) Biofilm surface. The longest part of the cell cluster was 1.26 ± 0.236 µm (Mean ± SD, *n* = 40, measured with ImageJ). (C) and (D) One‐cut face‐out SEM observation using an ultramicrotome. The arrowhead indicates the fibers observed between cells. The bidirectional arrow indicates the distance between cells and cavities within the biofilm. The black arrows in (D) indicate structures protruding at regular intervals from the cells. (E) Binarized SEM image (C) and analysis of the abundance of cells and cavities in the biofilm. The ratio of the area occupied by cells to that by cavities was 45.3:54.7 (calculated from the representative image shown in panel C). (F) Schematic diagram of the maturation process of a spongy biofilm.

**FIGURE 3:**
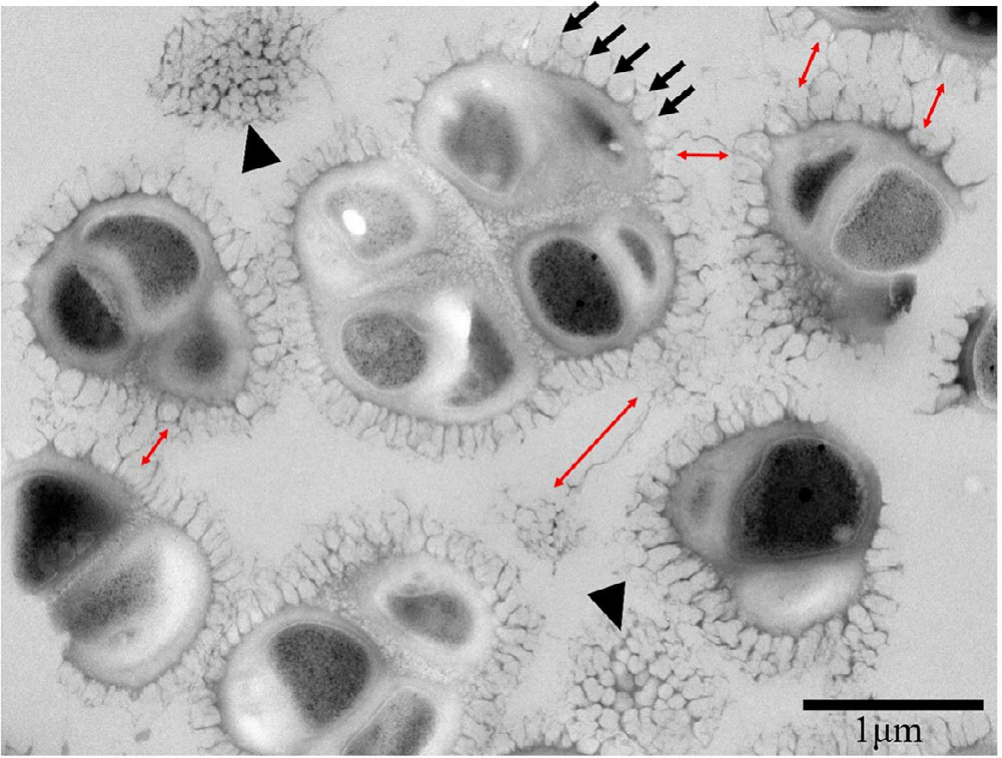
Capsule and cellular structure analysis using TEM. Arrows indicate structures arranged at regular intervals on the surface of the capsule. Arrowheads indicate the horizontal cross‐section of the capsule. The red double‐headed arrows indicate fibrous structures observed between cells.

**FIGURE 4:**
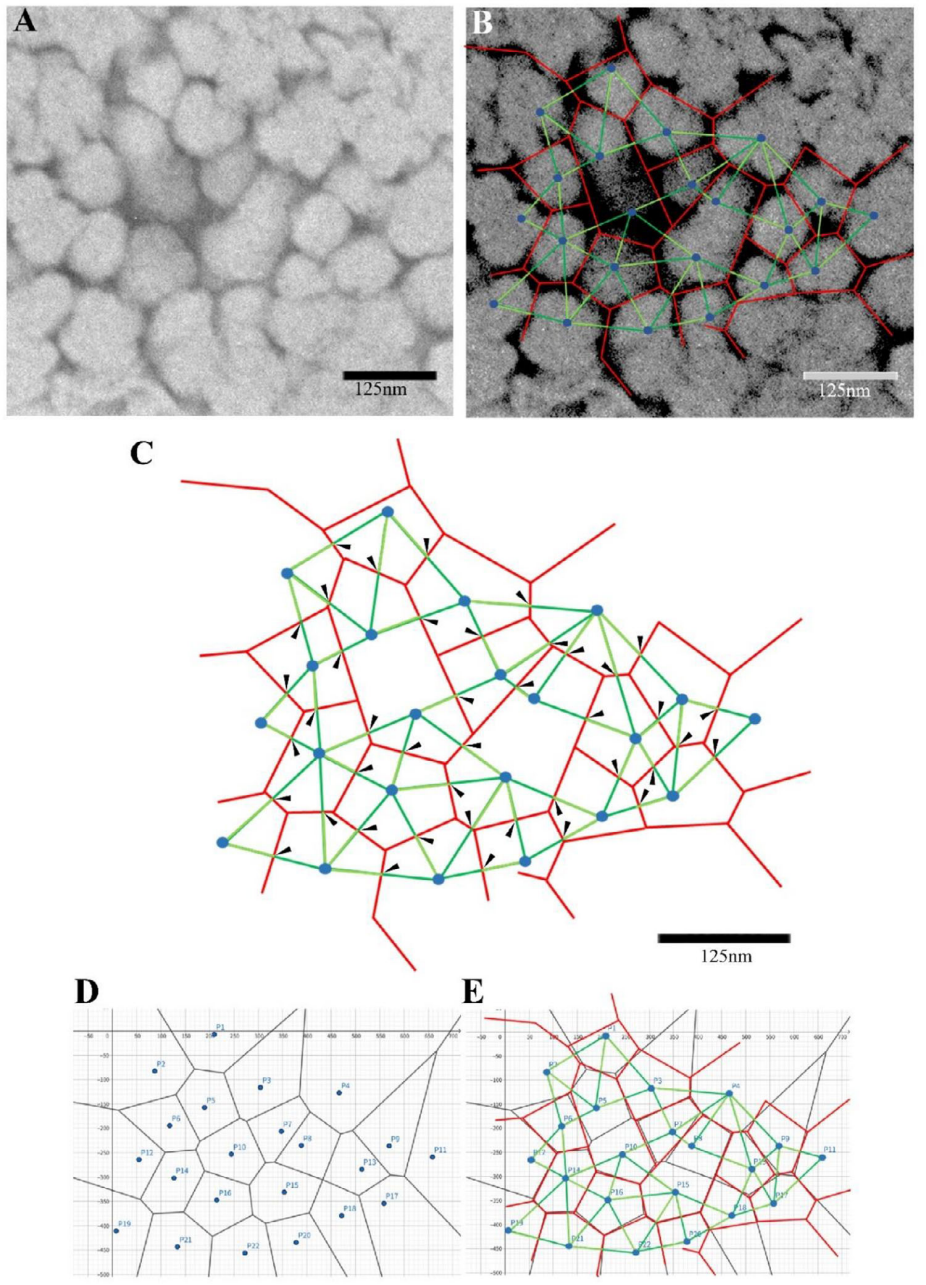
Vertical section of the capsule of *Micrococcus* sp. Clone 20, showing Voronoi structure. (A) Cross‐sectional TEM image of the capsule. (B) The walls forming the capsule are connected in the image by red straight lines (Voronoi edges), the Voronoi sites are depicted as blue points, and the Delaunay triangulation is represented by green lines. (C) The 42 arrowheads indicate the angles formed between the Voronoi edges (red) and the lines of the Delaunay triangulation (green), averaging 83.7° ± 5.99°. Data are shown as mean ± SD (*n* = 42 angles, descriptive statistics). (D) A Voronoi diagram created using GeoGebra. The Voronoi points predicted in (B) were converted to coordinates, and the diagram was derived based on these coordinates using the principle of distance minimization. (E) Comparison of the Voronoi diagram derived through calculations (black) and the one manually constructed through visual inspection (red) are shown, overlaid for comparison.

**FIGURE 5:**
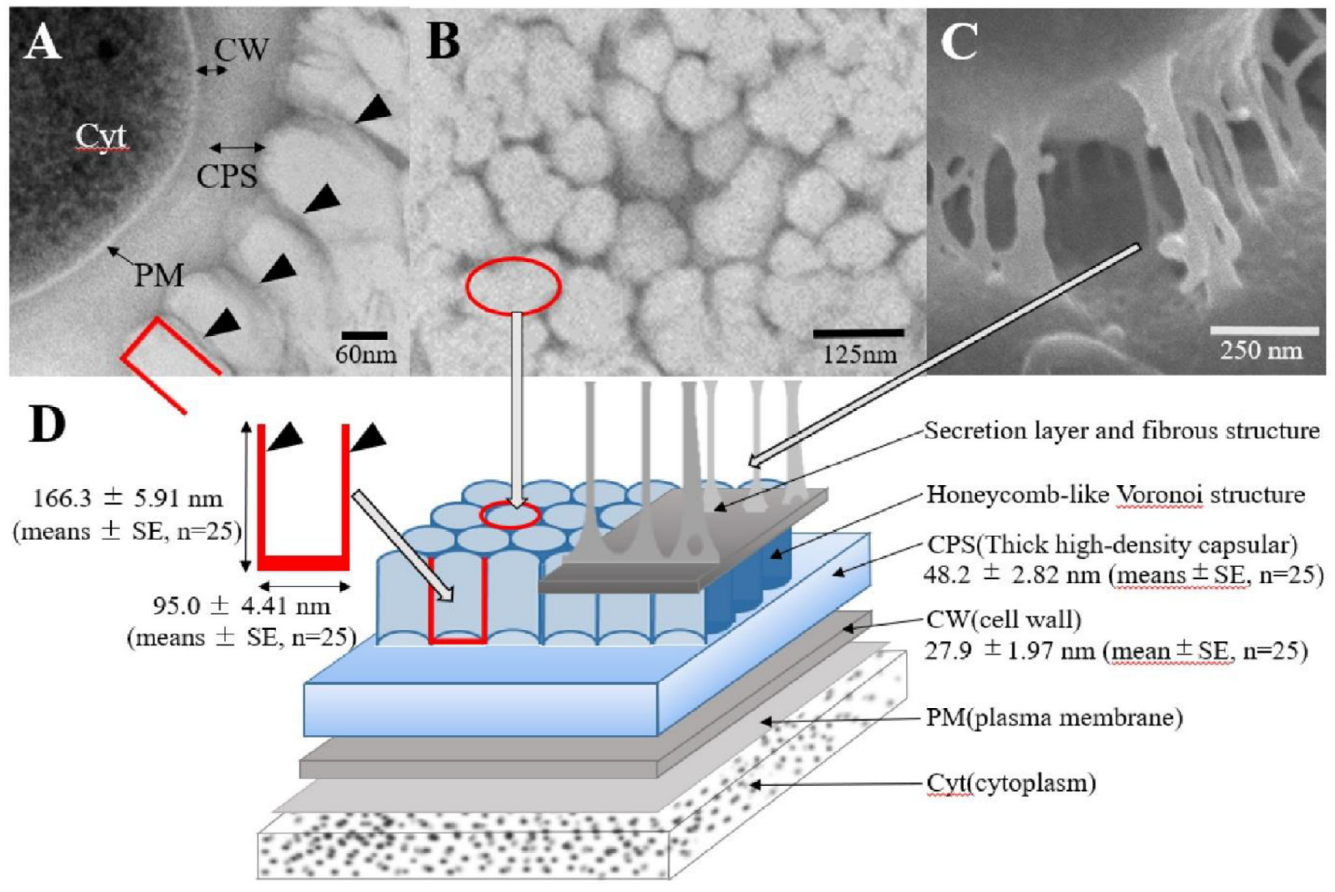
Structure of *Micrococcus* sp. Clone 20 cells and biofilms. (A) Cell cross‐sectional structure observed using TEM; Cyt, cytoplasm; PM, plasma membrane; CW, cell wall; CPS, capsular layer. Arrowheads indicate the honeycomb‐like Voronoi structure layers. (B) Honeycomb‐like Voronoi structure layer observed in the cross‐section of TEM. (C) Cell surface observed using SEM. (D) Schematic illustration of the structure of the cell and biofilm of *Micrococcus* sp. Clone20 in cross‐section. The red box indicates the partition walls that formed the honeycomb‐like structure.

SEM analysis of the cell surface revealed that the cells were spherical, characteristic of *Micrococcus* cells, with a size of 1.26 ± 0.236 µm (Mean ± SD, *n* = 40), indicating variability in diameter. In addition, a well‐known characteristic of *Micrococcus* was observed, namely that 1–4 cells often form clusters within a capsule. Fibrous material was observed between the cells, creating intercellular spaces (Figure 2A,B). SEM of cross‐sectional sections of cells showed that the intercellular spaces were scattered throughout the biofilm (Figure 2C,D). The white bidirectional arrows in Figure 2B,C indicate the intercellular spaces, whereas the red arrowheads in Figure 2B,D indicate fibrous structures observed between cells. In Figure 2D, the black arrows show cross‐sections of the capsule; although these structures appear as protrusions at this stage, TEM analysis (Figures 3, 4, and 5A,B) confirmed that they represent the edges of pore structures. SEM images of the biofilm cross‐section in Figure 2C were binarized, and the cell regions were extracted to determine the proportion of cells and spaces in the biofilm. The ratio of the area occupied by cells to that occupied by the cavities was 45.3:54.7 (Figure 2E). The two cells remain attached immediately after division; however, as the biofilm matured, fibrous secretions created spaces between the cells (Figure 2F). The red arrowheads in this schematic diagram correspond to the fibrous structures observed between cells in Figure 2B,D.

### 2.4 Cross‐Sectional Analysis of Cells and Capsules

The protrusions shown in Figure 2D were more clearly observed in TEM images; the black arrows in Figure 3 indicate these regularly arranged protrusions on the capsule surface. These protrusions were equally spaced around the perimeter of the cell, with gaps of 95.0 ± 4.41 nm (means ± SE, *n* = 25) (Figure 3). The length of these evenly spaced protrusions was 166.3 ± 5.91 (mean ± SE, *n* = 25) nm (Figure 3). The structures that appeared cut on a plane perpendicular to the spines had a shape similar to a honeycomb Voronoi diagram (Figure 3: arrowhead, and Figure 4A). Therefore, the spine‐like structures were concavities with a diameter and depth of 95.0 and 166.3 nm, respectively. In addition, long fibrous structures connecting cells were identified (Figure 3: red bidirectional arrows indicate fibrous structures).

### 2.5 Honeycomb‐Like Voronoi Structure of the Capsule Layer

Cells divide and alter their shape in response to their environment. In this study, images were obtained by preparing linear sections of spherical cells using an ultramicrotome. Therefore, distortions were more notable toward the periphery. Therefore, it is challenging to precisely determine Voronoi edges, Voronoi sites, perpendicular bisectors, and Delaunay triangulation through visual inspection. Irrespective of this, the observed porous structure of the capsule followed the principles of the Voronoi diagram. According to the Voronoi structure, the Voronoi edge (red) and the Delaunay triangulation line (green) intersect perpendicularly (Figure 4B).

Furthermore, the angles measured at 42 locations averaged 83.7° ± 5.99° (means ± SD, Relative error: 7.0%), whereas the theoretical value was 90° (Figure 4C).

Considering the standard deviation (SD) among the angles of 5.99°, some of the measured values ranged close to 90°. Furthermore, the Voronoi points predicted in Figure 4B were converted into coordinates, and a Voronoi diagram was created using GeoGebra based on the principle of distance minimization (Figure 4D). When the Voronoi diagram generated using GeoGebra was overlaid with the manually constructed diagram based on visual inspection, they generally overlapped (Figure 4E).

### 2.6 *Micrococcal* Cell, Capsule, and Extracellular Compartment

*Micrococcus* cells are composed of cytoplasm, a cell membrane, and a 27.9 ± 1.97 nm‐ (mean ± SE, *n* = 25) thick cell wall enveloped by the high‐density capsular layer with a thickness of 48.2 ± 2.82 nm (means ± SD), followed by a subsequent layer of honeycomb‐like Voronoi structures (166.3 ± 5.91 nm) (Figure 5A,D). In Figure 5D, the red box indicates the partition walls that form this honeycomb‐like structure. A significant quantity of secretions enveloped the cells and the capsular layer, with many fibers protruding and linking the cells (Figures 2A,B and 5C,D). These fibers contributed to maintaining intercellular spacing and creating cavities, thereby supporting the formation of the sponge‐like biofilm structure. The cells were embedded as one‐to‐four‐cell clusters within the sponge‐like structure formed by the honeycomb‐like Voronoi capsule and fibrous networks, thereby being positioned at a deeper distance from the external environment (Figure 5D).

### 2.7 Dynamics of Honeycomb‐Like Voronoi Structural Capsular Formation During Cell Division

**FIGURE 6:**
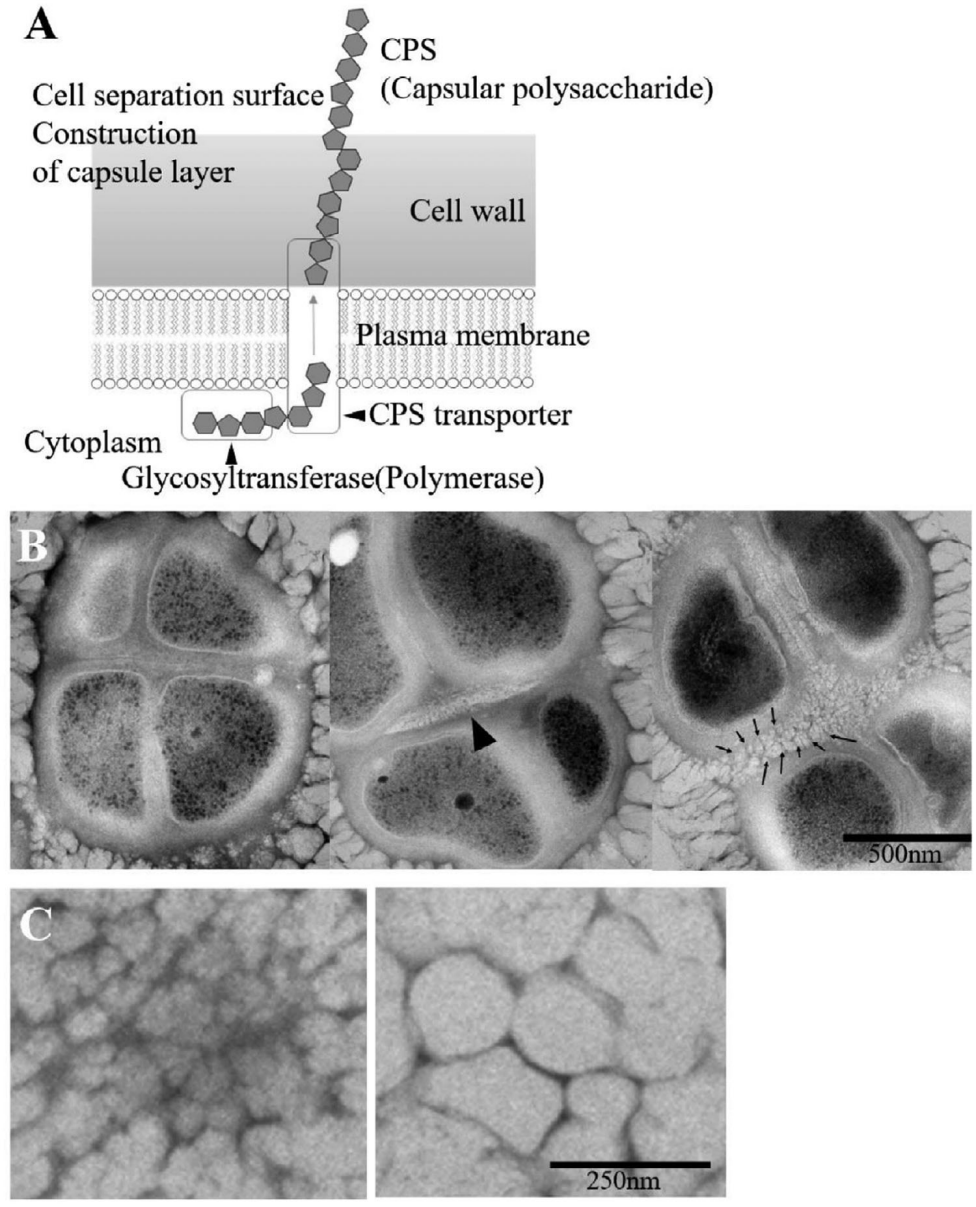
Dynamics of the honeycomb‐like voronoi structural capsule formation during *Micrococcus* sp. Clone 20 Cell Division, observed using TEM. (A) Schematic illustration of a molecular‐level diagram of CPS and capsule synthesis. (B) Synthesis of microfibers at the cell division interface. Arrowheads indicate the cleavage plane, and the small arrows indicate the beginning of the thorn‐like formation (horizontal cross‐section of honeycomb‐like Voronoi structural capsule). (C) Maturation of the honeycomb‐like Voronoi structural capsule (Vertical cross‐section) is shown in an estimated time series (from the left diagram to the right diagram). Figure 6A, adapted from Gao et al. [20], npj Biofilms and Microbiomes (2024), licensed under CC BY 4.0 (http://creativecommons.org/licenses/by/4.0/).

Figure 6A, adapted from Gao et al. [20], shows the molecular‐level synthesis of capsule sugar chains and other polymers within the cell and their subsequent release outside the cell. Using TEM, we observed the process in which the number of fibers that comprise the capsule gradually increases and assembles inside the capsule (Figure 6B). Figure 6B shows a horizontal cross‐section of the capsule, whereas Figure 6C presents a vertical cross‐section, providing a top‐view perspective of the pores. In Figure 6B, the arrowheads indicate the cleavage plane of cell division, and the small arrows mark the initiation of spine‐like protrusions associated with the honeycomb‐like Voronoi capsule. Fibrous material synthesis commenced immediately after cell division, with the surface rapidly becoming covered to create the dense capsular layer and the honeycomb‐like Voronoi structural layer (Figure 6B). As the process progressed, the number of finer fibers gradually increased and accumulated, creating walls that appeared to mature into a larger pore structure (Figure 6C).

### 2.8 Component Analysis of *Micrococcus* Colony and Copper Localization

**FIGURE 7:**
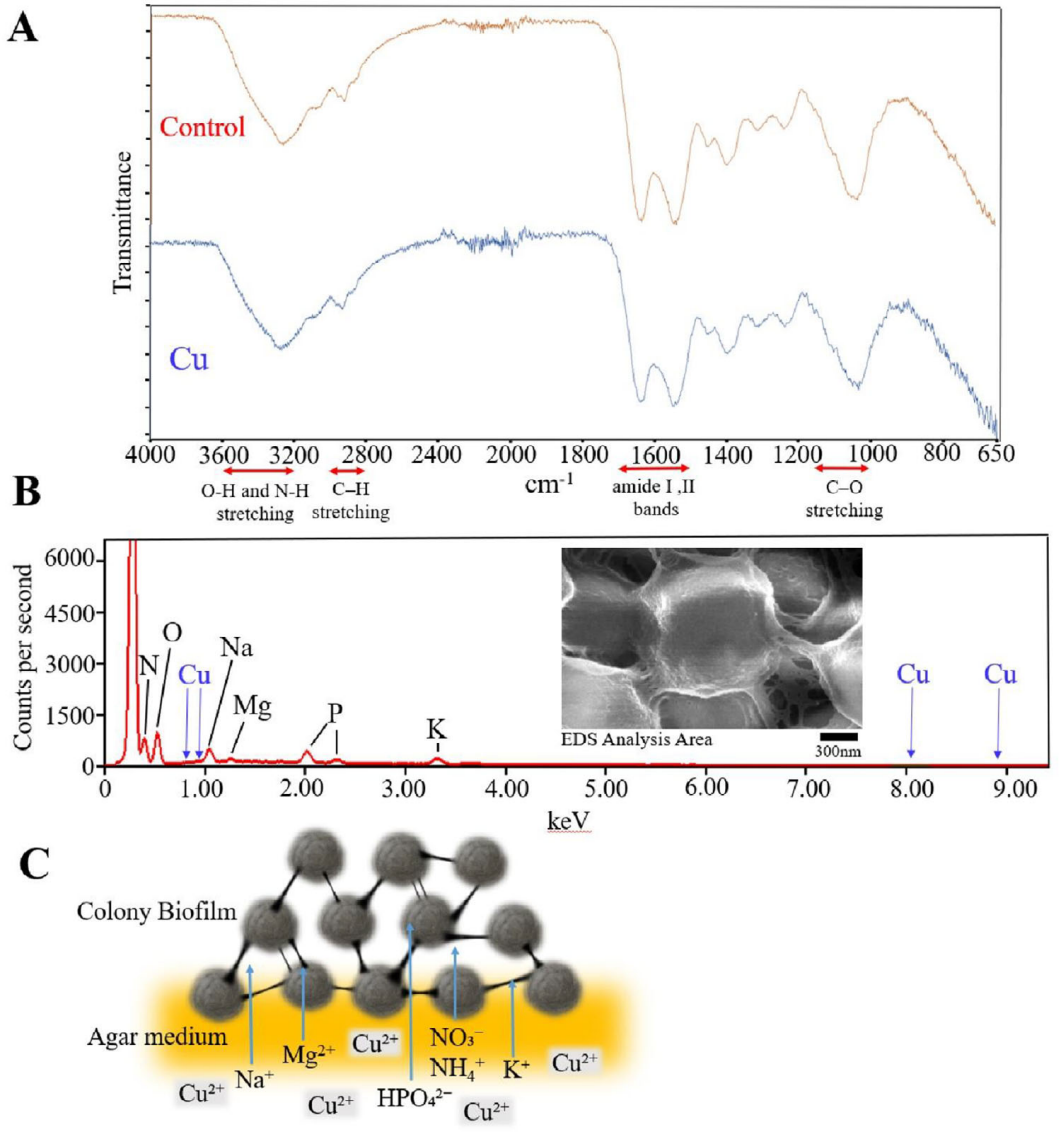
Cellular component analysis of *Micrococcus* sp. Clone 20. (A) Fourier Transform Infrared Spectroscopy analysis; a colony incubated in LB agar medium without copper and Cu^2+^ was used as the control: Colonies were incubated in LB with 1.2 mm copper (II) chloride agar medium for 5 d. A representative spectrum is shown. The areas indicated by the bidirectional arrows correspond to the O*─*H and N*─*H stretching vibrations (3200–3600 cm^−1^), the C*─*H stretching region (2800–3000 cm^−1^), the amide I and II bands (1500–1700 cm^−1^), and the C*─*O stretching vibrations (1000–1150 cm^−1^). (B) EDS analysis of cells incubated in LB and 1.2 mm copper (II) chloride agar medium for 5 d. SEM image showing the measurement area of the EDS analysis. (C) A model of selective ion absorption from the growth medium into the colony biofilm.

We analyzed the components of this particular *Micrococcus* colony using Fourier transform infrared (FT‐IR) spectroscopy and showed that the polysaccharide spectrum exhibited prominent peaks at 3200–3600 cm^−1^, representing the abundant hydroxyl groups (*─*OH), and at 1000–1150 and 1034 cm^−1^, which appeared to be C*─*O stretching vibrations. The prominent peak at 1034 cm^−1^ represents the ether bonds (C*─*O*─*C) of polysaccharides. A signal at 2928 cm^−1^, which likely represents a C*─*H stretching vibration (alkane group) of 2800–3000 cm^−1^, was also detected (Figure 6A). Three primary bands indicative of proteins were detected at 3267 cm^−1^ (N*─*H: amide A band), 1634 cm^−1^ (C = O, C*─*N: amide I band), and 1539 cm^−1^ (N*─*H, C*─*N: amide II band) (Figure 7A) [28].

Compared with those of the control (no copper addition to the agar medium), the peak areas of the amide I and II bands (1500–1700 cm^−1^) in the copper‐added group were reduced by approximately 19.2%. The peak area of the C–O stretching vibration (1000–1150 cm^−1^) was reduced by approximately 23.6% in the colonies grown on the copper‐added medium. Quantitative interpretation is challenging because absorbance varies based on measurement conditions and sample thickness; therefore, the peak intensity was presented as the relative area ratio of each functional group. The peak positions (wavelength values) indicate specific functional groups, and the appearance of new peaks reveals structural changes in the sample or differences in the bonding states of the functional groups. However, comparison of the copper‐added and nontreated samples revealed no significant peak shifts or emergence of new peaks (Figure 7A).

Elemental analysis of the *Micrococcus* colony cultured on copper‐treated Luria–Bertani (LB) agar medium was performed using EDS (Figure 7B). EDS detects Lα lines, which correspond to X‐rays emitted during the L‐series transitions of each element. Since the energy of Lα lines is lower than that of K‐series transitions, many elements exhibit Lα lines at relatively low energies. In the presence of copper, peaks corresponding to CuLI and CuLα transitions appear at approximately 0.93 keV, whereas CuKα and CuKβ peaks appear at 8.04 and 8.90 keV, respectively. However, none of these peaks were detected. Peaks corresponding to the Kα transitions of N (0.39 keV), O (0.52 keV), Na (1.04 keV), Mg (1.25 keV), and K (3.31 keV) were observed. Peaks corresponding to the Kα and Kβ transitions of P (2.01 and 2.13 keV, respectively) and O (0.52 and 0.57 keV, respectively) were also detected. Instead of the copper added to the medium, N, K, Mg, P, and Na were detected in the colony biofilm (Figure 7B). Figure 7C shows a model of selective ion absorption into the colony biofilm, showing retention of elements such as N, K, Mg, P, and Na, while excluding Cu.

## 3 Discussion

### 3.1 Characteristics of *Micrococcus* sp. Clone20 Isolated from Camphor Trees

The bacteria isolated from camphor leaves belonged to the genus *Micrococcus* (Figure 1A). However, instead of the characteristic yellow color of *Micrococcus* colony, the colony of the isolated Clone 20 was white (Figure 1B). *Micrococcus* typically forms yellow colonies owing to the production of diverse carotenoid pigments, such as echinenone (4‐keto‐β‐carotene), canthaxanthin (4’,4’‐diketo‐13‐carotene), and sarcinaxanthin [29, 30, 31]. These carotenoids are synthesized through biosynthesis of gene clusters (*crtE*, *crtB*, *crtI*, *crtE2*, *crtYg*, *crtYh*, and *crtX*) [32]. In Clone20, these gene clusters were either absent or nonfunctional.

In FT‐IR analysis, isoprenoids, the precursors of carotenoids, show C*─*H stretching vibration (alkene bonds = C*─*H) peaks in the region of 3000–3100 cm^−1^ and C═C stretching vibrations (conjugated double bonds) in the 1630–1640 cm^−1^ region. Our FT‐IR results showed that the two peaks overlapped with those of an O*─*H stretching vibration and a C═O vibration. The former produced a small signal, and the latter produced a strong peak but could not be distinguished from C═O (Figure 7A). Therefore, we were unable to determine the stage at which carotenoid biosynthesis was interrupted.

The discovery of a carotenoid synthesis gene‐deficient strain of *Micrococcus* provides insights into its metabolic mechanism and provides a useful model for studying the genetic basis of pigment production and its ecological significance in microbial adaptation to environmental stress. Moreover, carotenoids play important roles in photoprotection and oxidative stress tolerance; therefore, this strain can serve as a valuable tool to investigate the effects of the absence of pigments on microbial survival, plant–microbe interactions, and potential applications in biotechnology and environmental microbiology.

### 3.2 Structure and Formation of *Micrococcus* sp. Clone20 Biofilms

The cohesion, elasticity, and structure of *Escherichia coli* biofilms composed of amyloid curli fibers and cellulose have been extensively studied, including their concentric dome‐shaped ring architecture separated by deep fissures [33]. This biofilm structure, which functions as a bacterial community‐level mechanism, protects against the invasion of T7 phages [34]. Similarly, in *Bacillus subtilis*, amyloid fibers contribute to the structural integrity of biofilms [35]. In our FT‐IR results, sharp peaks were observed at 1634 and 1539 cm^−1^ (Figure 7A). Amyloids are rich in β‐sheet structures typically indicated by strong absorption in the amide I band (around 1610–1630 cm^−1^) derived from β‐sheets, as well as supplementary markers of β‐structure, such as N–H bending and C–N stretching in the amide II band (1530–1545 cm^−1^) [36, 37]. The biofilm matrix protein CdrA, which resembles a fibrous adhesion protein, has been identified in *Pseudomonas aeruginosa* [38]. These findings indicate that polysaccharides and proteins form biofilms with functional higher‐order structures. Consistent with this, the FT‐IR analysis results showed that the *Micrococcus* biofilm contained sugars and proteins (Figure 7A).

This study reveals previously uncharacterized morphological features of *Micrococcus*, including a sponge‐like biofilm and a honeycomb‐like Voronoi indentation structure in the capsular matrix. The biofilm of *Micrococcus* sp. Clone20 had several layers (Figure 5). The surface of the cells was covered with abundant secreted molecules that obscured the external shape (Figures 2A,B, and 5C).

The secretions exhibited fibrous structures that extended outward, appearing to connect the cells. However, whether the secretions connect the cells to prevent separation or to separate the cells remains to be determined. The fact that the spaces between the cells were maintained and cavities were formed indicates the latter (Figure 2F). Consequently, the resulting sponge‐like structure facilitates water and nutrient retention in the gaps, thus providing an adaptive advantage under fluctuating environmental conditions.

Ohmura et al. tracked cells in 3D *V. cholerae* biofilms and observed their anisotropic elastic and plastic responses to stress, as well as introducing a high‐resolution, noninvasive method to map mechanical properties correlated with polysaccharide distribution [39]. Depetris et al. clarified the relationship between patchiness, structure, and function in phototrophic biofilms, which form complex spatial patterns in rivers [40]. Such an adaptation is particularly important for microorganisms inhabiting leaf surfaces. The camphor tree leaf surface is constantly exposed to wind, sunlight, and rain, leading to alternating cycles of dryness and excessive moisture. Therefore, *Micrococcus* may have evolved into sponge‐like biofilm structures or developed hybrid capsule structures with Voronoi‐like indentations and dense layers to survive in this harsh environment. These structures may have ultimately evolved as a bacterial self‐defense mechanism against heavy metals, such as copper. Unlike the conventional view, where bacteria are isolated from contaminated soils and acquire various functions to protect themselves from heavy metals, this may represent a function acquired through a different evolutionary process.

Beneath the surface, depressions of 95.0 ± 4.41 nm (means ± SE, *n* = 25) in diameter and 166.3 ± 5.91 nm (means ± SE, *n* = 25) in depth appeared side‐by‐side without gaps, forming a honeycomb‐like Voronoi structure (Figures 2 and 5). Further inside the layer of the honeycomb‐like Voronoi structures, a thick, high‐density capsule layer was observed (Figure 5A,D). The honeycomb‐like Voronoi structural layer and the thick, high‐density capsule layer were formed by combining fine fibrous material from the beginning of cell division (Figure 6B).

Numerous studies have investigated the adsorption and accumulation of heavy metals in *Micrococcus* [2, 11, 12, 41, 42]. The extracellular matrix of *Micrococcus* primarily consists of extracellular polysaccharides [43, 44]. *Micrococcus luteus* DE2008 biosorbs lead and copper within its extracellular polysaccharide envelope, without intracellular accumulation [1]. Using TEM and SEM/EDS, Letnik et al. revealed that copper accumulates on the bacterial surface and intracellularly [4]. The same observation was made in our study: the hydroxyl, amino, and carboxyl groups were abundant in the biofilm (Figure 7A).

Colonies cultured in a medium containing copper showed a decrease in the peak areas of amino I and II bands and C–O stretching vibration peaks (Figure 7A). The copper in the medium may have interacted with the polysaccharides and proteins in the biofilm. However, no significant peak shifts were observed. FT‐IR peak areas can vary with sample placement or humidity. Therefore, these results alone are insufficient to determine copper binding. Therefore, we used EDS analysis to further examine copper localization within the biofilm. EDS analysis revealed that *Micrococcus* selectively takes up essential N, P, K, and Mg ions from the medium into the biofilm, while blocking the uptake of unwanted metals, such as copper, into the cells (Figure 7B). We hypothesized that the multiple layers with the honeycomb‐like Voronoi structure facilitate the ability to limit the uptake of heavy metals and selectively incorporate the necessary ions into the biofilm (Figure 7C). In addition to metal resistance, *Micrococcus* is also adapted to other extreme stresses. For example, *Micrococcus luteus* (V017) is resistant to radiation [45], and possesses a system that enables survival in extremely harsh environments. Thus, it may have a multilayered defense system against external stresses, including the honeycomb‐like Voronoi structural layer. The synthesis of the honeycomb‐like Voronoi structure revealed in this study begins upon cell division and the formation of a cleavage plane, indicating a potential mechanism for cellular self‐protection against environmental factors (Figure 6B). The size of the holes in the structure may increase as the fibers are pulled apart, and neighboring fibers may be reorganized as the fine fibers become densely entangled at the splitting surface and grow to form small holes (Figure 6C). Covering cells with a concave structural layer may mitigate local stress and ensure stability by evenly distributing external pressure and tensile forces. The concavities may also provide room for deformation, enabling the cells to respond flexibly to compression and tension, and elastic and plastic deformation may also be evenly distributed. Notably, a significant amount of energy is required to produce large quantities of the constituent compounds needed to create thick walls, such as the capsular layer of *Micrococcus*.

The placement of recesses can reduce the amount of material used, while maintaining strength, thereby enabling an energy reduction. In particular, when using self‐assembly and Voronoi structures, spaces can be filled efficiently, thus creating stronger structures with less material. A hybrid structure consisting of a thick high‐density capsule layer and a honeycomb‐like Voronoi structural layer (Figure 5A,D) can also achieve fluid transport and strength. This structural strategy is important because *Micrococcus* sp. Clone20 encounters the challenge of securing nutrients on the surface of camphor tree leaves (Figure 1).

In this study*, Micrococcus* sp. Clone20 selectively absorbed essential metal ions from the medium into the biofilm, while limiting the uptake of harmful heavy metals, such as copper (Figure 7B,C). Moscoso et al. observed the ultrastructure of *Streptococcus pneumoniae* R6 biofilms and reported large void spaces in transverse sections of the biofilm. These void spaces were originally filled with water and may be important for nutrient supply and waste removal [46].

In contrast to these void‐like spaces, the sponge‐like biofilm and honeycomb‐like capsule structures identified in *Micrococcus* may represent a distinct strategy for balancing nutrient retention and heavy metal exclusion. In summary, the discovery of these structures in *Micrococcus* provides a previously unrecognized perspective on microbial survival strategies under fluctuating environmental conditions. These findings expand our understanding of physical structural adaptations that support microbial persistence and reveal potential applications in agriculture, where biofilm‐mediated water and nutrient retention may improve soil fertility and plant stress tolerance. In addition, this study highlights a distinct evolutionary pathway of heavy metal avoidance that differs from conventional adsorption‐based mechanisms. Future studies should investigate the genetic basis and regulatory networks determining the formation of these structures to enhance the understanding of their ecological and biotechnological significance.

These findings may also serve as a theoretical framework for the development of novel bioremediation strategies, particularly in environments contaminated with heavy metals, where structural avoidance mechanisms may complement or enhance conventional adsorption‐based approaches.

### 3.3 Role of Biofilm and Capsule Structures in Copper Resistance in *Micrococcus*: Insights into Exclusion Mechanisms

In this study, the structure of the biofilm and capsule formed by *Micrococcus* was closely associated with its high resistance to copper. The biofilm formed by *Micrococcus* consists of a hybrid structure that combines dense and porous layers, with a thick capsule presenting a honeycomb‐like Voronoi structure (Figures 2 and 5). This complex 3D structure functions as a physical barrier that impedes the diffusion of metal ions. Stewart showed that the diffusion of metal ions follows Fick's law and that, in biofilms, the time required for metal ions to reach 90% of their concentration at the bottom or center of the biofilm increases in proportion to the square of the diffusion distance [17]. Based on this knowledge, the spongy structure of the *Micrococcus* biofilm, which increases the intercellular distance, and the thick capsule containing pores further prevent the entry of Cu^2^⁺ into the biofilm. In this study, the copper resistance ability was significantly higher in the biofilm state compared with that of the planktonic cells (Figure 1B,C), supporting the idea that these structures aided in blocking Cu^2^⁺ penetration (Figure 7).

Similar phenomena have been reported in other microorganisms. For example, *Pseudomonas aeruginosa* biofilm exhibits 2–600‐fold higher resistance to heavy metals compared with that of planktonic cells [16], consistent with the copper resistance ability of *Micrococcus* observed in this study. These findings indicate that the unique structural characteristics of the biofilm and capsule in *Micrococcus* act as a combination of physical barriers to diffusion and chemical adsorption/exclusion mechanisms, preventing Cu^2^⁺ from entering the cells and contributing to its high resistance. This highlights the importance of structural analysis of biofilms in elucidating microbial metal resistance mechanisms [47].

Furthermore, the extracellular polymeric substances (EPS) and extracellular DNA (eDNA), which constitute the biofilm, play critical roles. The complexes formed by EPS and eDNA possess a negative charge and function as “electrostatic traps” by attracting cations, such as heavy metals, while preventing their internal diffusion [48]. In addition, EPSs form nanoscale pores, which provide a molecular sieve effect because of the larger hydration radius of metal ions, thereby restricting their movement within the biofilm [49]. Because of the hydration radius of Cu^2^⁺ is approximately 4.19 Å, its movement is likely significantly restricted by the pore structure of EPSs.

Our study introduces a previously unrecognized concept of heavy metal avoidance based on physical structural strategies, thereby expanding the current understanding of microbial resistance mechanisms and revealing potential applications in environmental resilience. Future studies should investigate the genetic and regulatory mechanisms underlying the formation of these unique capsules and biofilm structures to clarify their ecological roles under heavy metal stress.

### 3.4 Elemental Composition Retained in *Micrococcus* Biofilms: Implications for Material Cycling and Ecological Symbiosis

Our results revealed that *Micrococcus* sp. Clone20 retained N, P, K, and Mg in its biofilm, which could serve as a stable supply of these elements for plants. Previous studies have shown a potential symbiotic relationship between *Micrococcus* species and plants, as *Micrococcus* sp. NII‐0909 is capable of phosphate solubilization and produces IAA [5], *Micrococcus aloeverae* DCB‐20 has unique potential for plant hormone IAA production [14], and *Micrococcus luteus* MlS14 promotes the growth and stress tolerance in *Arabidopsis thaliana* [7]. Furthermore, the microbiomes of individual plant leaves can regulate the genetic control network of the plant and promote leaf growth [50]. Therefore, *Micrococcus* sp. Clone20 may form a similar symbiotic relationship with plants.

Our EDS analysis detected phosphorus in the biofilm of *Micrococcus* (Figure 7B). *Micrococcus* secretes eDNA, which is essential for maintaining the biofilm structure [51]. Moreover, salt‐tolerant *Micrococcus* varians subsp. *halophilus* produces an extracellular 5’‐nucleotidase that requires 2 m NaCl or 2.5 m KCl and 0.1 mm Co^2^⁺ or Mn^2^⁺ for maximum activity [52]. Our elemental analysis using EDS revealed that the biofilms were rich in Na and K (Figure 7B). *Micrococcus* sp. NII‐0909, found in forest soil, is a phosphate‐solubilizing bacterium [17], and may be important in environmental phosphorus cycling. Our findings reveal that the *Micrococcus* strains detected in this study may contribute to phosphorus accumulation and biofilm maintenance on the surface of plant leaves through eDNA secretion and nucleotidase enzyme activity. However, a limitation of this study is that the functional roles of eDNA secretion and nucleotidase activity were inferred from genomic or literature data and not directly confirmed through molecular or biochemical assays. Therefore, further studies should verify the actual enzymatic activity and gene expression involved in phosphorus cycling and biofilm maintenance in situ.

## 4 Conclusion

This study provides valuable insights into the copper resistance mechanisms of *Micrococcus* isolated from camphor trees, with a focus on its unique biofilm and capsule structures. Its sponge‐like biofilm structure, formed by fibrous materials that create intercellular spaces, and the hybrid capsule structure, consisting of a honeycomb‐like Voronoi pattern and a dense layer, play crucial roles in protecting the cells from external stress and controlling the intrusion of harmful substances such as Cu^2+^. Notably, the ability of the biofilm to selectively absorb essential ions, while limiting the entry of harmful ions reveals its importance in environmental adaptability and survival. Moisture and nutrient retention by the capsule layer, along with its potential role in plant symbiosis, highlight the ecological significance of *Micrococcus* in nature. Through bacterial symbiosis with plants, *Micrococcus* has evolved to adapt to harsh environments, such as dryness or excessive moisture on leaf surfaces, by acquiring specialized biofilm and capsule structures. These findings provide novel insights into the symbiosis between plants and bacteria, reveal important challenges, provide novel perspectives on the use of *Micrococcus* in bioremediation, and contribute to the advancement of conventional strategies for purifying heavy metal‐contaminated water through its ability to adsorb and accumulate heavy metals.

This study has some limitations and raises important future questions. The experiment was restricted to Cu^2+^ stress under laboratory conditions, which may not fully represent the broad range of selective pressures that microbes face on leaf surfaces. Moreover, the present analysis was based on isolates obtained from a single camphor tree at one location, which limits the generalizability of our findings. To enhance the evaluation of the ecological and evolutionary significance of these biofilm and capsule structures, *Micrococcus* strains should be isolated from multiple camphor trees and diverse environments and subjected to comparative analysis. In addition, microbes on leaves exhibit extraordinarily high tolerance to heavy metals, a trait whose biological significance remains unclear and should be addressed further. Moreover, whether the observed honeycomb‐like capsules and sponge‐like biofilms represent specific evolutionary responses to Cu^2+^ or more general adaptations to harsh environments remains unclear. To address these questions, future studies should compare the role of copper with other heavy metals and environmental factors. Addressing these issues will provide key insights into determining whether metal ions have played a proactive role in driving microbial evolution or whether extreme environments have facilitated the development of specialized microbial structures.

## 5 Experimental Section

### 5.1 Isolation and Identification of Bacteria from Camphor Trees

Leaves from a mature *Cinnamomum camphora* tree located in Fukui City, Japan, were sampled in October 2024. Several mature leaves without visible disease symptoms were collected, placed in a sterile plastic bag (Ziploc, Asahi Kasei, Tokyo, Japan), and supplemented with sterile distilled water. Then, the wash solution was spread onto LB agar medium for bacterial isolation. Copper‐resistant bacteria on the surface of camphor trees were screened using LB (Lennox, Nacalai Tesque, Kyoto, Japan; code: 20066‐95) and agar (Nacalai Tesque; code: 01162015) media with 40 µm copper (II) chloride (Nacalai Tesque; code: 09505‐85). Colonies were obtained following incubation at 20°C for 7 d and were amplified using direct colony polymerase chain reaction with the primers 27F (5’‐AGAGAGTTTGATCCTGGCTCAG‐3’) and 1492R (5’‐GGCTACCTTGTTACGACTT‐3’) for the 16S rDNA region [53, 54]. The PCR products were subjected to agarose gel electrophoresis, extracted, and purified. Sequencing reactions were performed using the BigDye Terminator v3.1 Cycle Sequencing Kit (Applied Biosystems, Foster City, CA) on an Applied Biosystems 3730xl DNA Analyzer (Applied Biosystems).

National Center for Biotechnology Information (NCBI) Basic local Alignment Search Toom (BLAST) was used to identify bacteria with the predetermined 1395‐base sequence (Accession No. PV202384). 16S‐rDNA sequences were obtained for the top 50 most similar species, including 19 *Micrococcus*, *Citricoccus*, and *Arthrobacter* strains: *Micrococcus flavus* LW4 (NR043881), *Micrococcus aloeverae* AE‐6 (NR134088), *Micrococcus yunnanensis* YIM 65004 (NR116578), *Micrococcus luteus* DSM 20030 (NR037113), *Micrococcus endophyticus* YIM 56238 (NR044365), *Micrococcus cohnii* WS4601 (NR117194), *Micrococcus antarcticus* T2 (NR025285), *Micrococcus lylae* DSM 20315 (NR026200), *Citricoccus alkalitolerans* YIM 70010 (NR025771), *Citricoccus nitrophenolicus* PNP1 (NR117546), *Citricoccus yambaruensis* PS9 (NR132666), *Citricoccus massiliensis* Marseille‐P4330 (NR179647), *Citricoccus parietis* 02‐Je‐010 (NR104498), *Citricoccus zhacaiensis* FS24 (NR116270), *Arthrobacter pokkalii* P3B162 (NR149802), *Arthrobacter globiformis* JCM 1332 (NR112192), *Arthrobacter pascens* DSM 20545 (NR026191), *Arthrobacter halodurans* JSM078085 (NR116375), and *Arthrobacter rhombi* F98.3HR69 (NR026448) [14]. The isolated Clone20 (PV202384) was also included. All data were stored in the FASTA format [55, 56].

Phylogenetic tree construction was performed using MEGA 12 (www.megasoftware.net, Philadelphia, PA) [57], with the minimum evolution method (neighbor‐joining method) [58, 59]. The Kimura 2‐parameter model was selected as the evolutionary model, assuming uniform evolutionary rates among the lineages [60]. The ME Search Level was set to medium to balance calculation precision and efficiency during the analysis. Parallel processing was performed using four threads to accelerate the computation. Pairwise deletion was applied to address gaps and missing data; missing data were ignored for each sequence pair during the analysis. Bootstrap analysis was performed with 1000 replicates to assess the confidence of the tree [61].

### 5.2 Colony Observation and Passaging Culture

Colonies were collected using a sterile stick and lightly spread on the surface of LB agar plates, either supplemented with 1.2 mm copper (II) chloride dihydrate or without supplementation, while partially preserving the biofilm structure. The plates were incubated at 20°C for 5 d and subsequently photographed using a Canon digital camera (G7X; Canon Inc., Tokyo, Japan).

Separately, colonies were suspended in liquid medium by vortexing for 2 min using a vortex mixer (Taitec Co., Ichinomiya, Aichi, Japan), and the suspension was adjusted to an OD_600_ of 0.2. An aliquot (25 µL) of this suspension was inoculated into test tubes containing LB liquid medium (5 mL) and shaken at 170 rpm to disrupt the biofilm structure, thereby generating planktonic cells for the copper tolerance assay.

The assay was conducted in LB liquid media supplemented with copper (II) chloride dihydrate (0.26, 0.52, or 1.2 mm), as well as in unsupplemented medium. Cultures were incubated at 20°C for 40, 48, and 72 h. After incubation, samples were transferred to plastic cuvettes with a 0.5 cm light path, and absorbance at 600 nm was measured using a spectrophotometer (UVIDEC‐340, JASCO, Tokyo, Japan) to estimate cell density.

### 5.3 Cell Sample Preparation for Electron Microscopy and EDS Analysis

Cells were incubated in LB (Lennox) and copper (II) chloride (1.2 mm) agar medium at 20°C for 5 d under aerobic conditions. The colonies were removed, sandwiched between pure gold plates, and flash‐frozen in liquefied propane (−175°C). Subsequently, a solution containing 2% glutaraldehyde in ethanol and 2% distilled water was added and incubated at −80°C for 48 h.

This was followed by freeze‐displacement treatment. The temperature was increased incrementally: 3 h at −20 °C and 3 h at 4 °C. Further treatment with anhydrous ethanol was conducted thrice for 30 min each at 25 °C. Finally, the samples were incubated overnight in anhydrous ethanol.

### 5.4 Cell Surface Observations using SEM

After pretreatment, the cells were incubated in a 5:5 mixture of anhydrous ethanol and *t*‐butyl alcohol for 1 h at room temperature, followed by three treatments with *t*‐butyl alcohol for 30 min. Following freeze treatment, they were vacuum dried at 4 °C. This sample was used for cell surface observation. A conductive carbon coating was applied, and the cells were observed and photographed using SEM (JSM‐7500F; JEOL Ltd., Tokyo, Japan) at an acceleration voltage of 3.0 kV. The samples prepared in this process were also used for EDS elemental analysis.

### 5.5 Cross‐Sectional Observation of Cells using SEM and TEM

After pretreatment, propylene oxide (PO) treatment was repeated twice for 30 min at room temperature, followed by incubation with a 7:3 mixture of PO and resin (Quetol‐812; Nisshin EM Co., Tokyo, Japan) for 1 h at room temperature. PO was fully volatilized overnight, followed by the addition of Quetol‐812 and incubation at 60°C for 48 h to complete resin embedding. Embedded samples were sliced once with an ultramicrotome diamond knife (Ultracut UCT; Leica, Vienna, Austria), coated with conductive carbon, and examined and photographed using SEM (JSM‐7500F; JEOL Ltd.) at 3.0 kV.

For TEM, the embedded samples were sliced into 70 nm sections with an ultramicrotome diamond knife, placed on a copper grid, treated with 2% uranyl acetate for 15 min at room temperature, and stained with lead stain solution (Sigma‐Aldrich Co., St. Louis, MO) for 3 min at room temperature for electron staining. Observations were made using TEM (JEM‐1400Plis; JEOL Ltd.) at an acceleration voltage of 100 kV. Photographs were captured with a CCD camera (EM‐14830RUBY2; JEOL Ltd.).

### 5.6 Measurement of Area and Distances using Images

Image analysis was performed using ImageJ (National Institutes of Health, Bethesda, MD). For images with low contrast that hindered clear identification of cell boundaries, preprocessing was conducted to enhance segmentation.

Edge detection was performed to emphasize regions with sharp intensity transitions, followed by a Gaussian blur to reduce noise and smooth contours. Thresholding was used to convert the images to a binary format and clearly separate cells from the background. In the resulting binary images, cell regions were inverted and colored in black against a white background to create fully segmented images. The proportion of the image area occupied by black regions (cellular areas) was calculated using the area measurement function of the software, thus enabling quantitative evaluation of cell distribution and density.

### 5.7 Mathematical Definition of Voronoi Diagram

A Voronoi diagram partitions the plane based on the Euclidean distance to a specific set of seed points. For each seed point *Pi*, the corresponding Voronoi region *V* (*Pi*) is defined as follows:


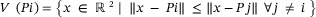

where || *x* ‐ *Pi* || is the Euclidean distance between point xxx and the seed point *Pi*. Point xxx belongs to the region *V* (*Pi*) if, and only if, it is closer to *Pi* than to any other point, *Pj*. Voronoi diagrams are constructed geometrically by computing perpendicular bisectors between pairs of points and intersecting the corresponding half‐planes to form regions. [25].

### 5.8 Construction of Voronoi Diagrams of the Porous Capsule Cross‐Section

The Voronoi edges were manually determined through visual inspection, and all perpendicular bisectors between pairs of points were drawn by hand. The intersection points of these perpendicular bisectors were considered Voronoi sites. The triangles formed by the perpendicular bisectors were regarded as the Delaunay triangulation. The angles between the Voronoi edges and the lines forming the Delaunay triangulation were measured using ImageJ. The Angle Tool was used to manually select and measure the angles at the intersection points of the Voronoi edges and the Delaunay triangulation lines. Overall, 42 angles were measured, and the results were analyzed based on the mean and SD (variance), and compared with the theoretical values. A Voronoi diagram was generated using GeoGebra (https://www.geogebra.org/, GeoGebra GmbH, located in Linz, Austria) based on the coordinates of seed points determined from hand‐drawn diagrams. The obtained coordinates (points P1–P22) were entered into the Algebra View of GeoGebra. The coordinates used were as follows:

P1 = (209.57, −7.127), P2 = (87.129, −82.243), P3 = (304.57, −116.127), P4 = (466.57, −127.127), P5 = (189.57, −157.127), P6 = (117.57, −194.127), P7 = (347.57, −206.127), P8 = (388.57, −235.127), P9 = (569.57, −236.127), P10 = (244.57, −253.127), P11 = (658.57, −259.127), P12 = (54.57, −264.127), P13 = (513.129, −284.243), P14 = (126.57, −302.127), P15 = (353.57, −331.127), P16 = (214.57, −347.127), P17 = (558.57, −354.127), P18 = (471.57, −379.127), P19 = (7.548, −410.596), P20 = (378.129, −434.243), P21 = (133.57, −443.127), and P22 = (272.57, −456.127).

### 5.9 EDS Elemental Analysis

EDS elemental analysis (JED‐2300F; JEOL Ltd.) was performed on samples that had been fixed, dehydrated, dried, and carbon‐coated.

### 5.10 FT‐IR Analysis of Biofilm Components

Colonies were incubated on LB (Lennox) agar without copper and LB agar with copper (II) chloride (1.2 mm) at 20°C for 5 d under aerobic conditions. The colonies were removed, dried, and subjected to FT‐IR analysis (Spotlight 200; PerkinElmer Inc., Waltham, MA). The effects of copper addition on the biofilm were evaluated in terms of O–H and N–H stretching vibrations (3200–3600 cm^−1^); amide I and II bands (1500–1700 cm^−1^); and C–O stretching vibrations (1000–1150 cm^−1^). The peak areas in each transmittance region of the FT‐IR spectra were calculated. Two control experiments (without copper) and one copper‐supplemented experiment were conducted. The C–H stretching region (2800–3000 cm^−1^), which is unaffected by copper ions, was used as a reference (d), and the relative ratios of each peak area (a/d, b/d, c/d) were used as evaluation indices. A peak shift was observed owing to the addition of copper ions.

### 5.11 Statistical Analysis

All quantitative data are presented as mean ± SD or mean ± standard error (SE), as specified in the corresponding figure legends. The number of independent experiments or measurements (*n*) is indicated in each legend.

Copper tolerance assays (Figure 1B,C) were performed in triplicate and expressed as mean ± SE. Statistical significance between two groups was evaluated using Student's *t*‐test assuming equal variance (two‐tailed). Statistical significance was set at *p* < 0.05.

For image‐based analyses, the major axis length (longer of the vertical and horizontal dimensions) of individual cells (Figure 2A,B) was determined with 40 cells using ImageJ (National Institutes of Health, Bethesda, MD) and expressed as mean ± SD. The ratio of cell and cavity areas in the biofilm (Figure 2E) was calculated from the representative binarized SEM image shown in panel (C).

For Voronoi structural analysis (Figure 4C), 42 angles were measured and summarized as mean ± SD (*n* = 42 angles, descriptive statistics only, no hypothesis testing).

FT‐IR spectra (Figure 7A) are presented as representative examples to illustrate the spectral features observed under copper‐supplemented and nonsupplemented conditions.

EDS (Figure 7B) was used to identify elemental signals in cells cultured on copper‐supplemented agar medium. Representative spectra are shown to indicate the detection of copper and other elements; no statistical hypothesis testing was applied.

Image processing and morphometric analyses were performed using ImageJ, and Voronoi diagram construction was performed using GeoGebra (GeoGebra GmbH, Linz, Austria).

### 5.12 Ethical Approval

This study did not require formal ethical approval, as it involved no human or animal subjects. All aspects were conducted in strict accordance with the Code of Conduct for Researchers of the National Institute of Technology (KOSEN) and the guidelines of the Ethics Committee of the National Institute of Technology, Fukui College.

## Author Contributions

All authors contributed to the conception and design of the study. Data preparation, data collection, and analysis were performed by Toshiyuki Kawamura, Yui Naito, Yuji Yanagihara, Yosyun Onishi, Rajesree A/P Sivakumaran, and Eiki Matsui. The first draft of the manuscript was written by Toshiyuki Kawamura, and all the authors have commented on previous versions of the manuscript. All authors have read and approved the final version of the manuscript.

## Funding

This project was supported by the Nakatani Foundation for Advancement of Measuring Technologies in Biomedical Engineering of Japan (Grant number: 23P08).

## Conflicts of Interest

The authors declare no conflicts of interest.

## Data Availability

The data that support the findings of this study are available from the corresponding author upon reasonable request.
